# High-Performance Triboelectric Nanogenerator Based on PVDF Nanofibers Modified by a Charge Control Agent n-Propyl Gallate

**DOI:** 10.3390/ma18133089

**Published:** 2025-06-30

**Authors:** Chao Li, Xueying Yang, Xin Tang, Ying Yang, Linjiang Shen, Dawei Gu, Mustafa Eginligil

**Affiliations:** 1 Department of Physics, School of Physical and Mathematical Sciences, Nanjing Tech University, Nanjing 211816, China; ngbplc@njtech.edu.cn (C.L.); 202161221025@njtech.edu.cn (X.Y.); tangx0301@njtech.edu.cn (X.T.); yingyang@njtech.edu.cn (Y.Y.); ljshen@njtech.edu.cn (L.S.); 2 State Key Laboratory of Flexible Electronics (LoFE), School of Flexible Electronics (Future Technologies) & Institute of Advanced Materials (IAM), Nanjing Tech University, Nanjing 211816, China

**Keywords:** PVDF, nanofiber, n-propyl gallate, β phase, energy harvesting

## Abstract

Triboelectric nanogenerators (TENGs), as an emerging energy harvesting device, can efficiently convert the weak mechanical energy in the environment into electrical energy, demonstrating significant potential in self-powered systems. In this study, polyvinylidene fluoride (PVDF) nanofiber films mixed with a small amount of n-propyl gallate (PG) were prepared by using the electrospinning technique, and TENGs were fabricated based on these films. Unexpectedly, experimental results showed that PG (with 0.5–2.5 wt%) did not affect the β phase of the PVDF. However, the TENG based on PVDF/PG composite nanofiber film with 1 wt% PG (PG1-TENG) exhibited large output values of 334 V, 4.36 μA, and 78.4 nC for output voltage, current, and transferred charge, respectively, with a power density of 5.27 W/m^2^, which highlights ~60% improvement in output voltage over pristine PVDF-TENG. This observation was attributed to the unique charge regulation ability of PG, without altering PVDF’s β phase. Furthermore, application potential of PG1-TENG was demonstrated by powering up an LCD calculator and 480 LEDs.

## 1. Introduction

Triboelectric nanogenerators (TENGs) based on electro-spun nanofibers are promising for wearable electronic applications [1]. Polyvinylidene fluoride (PVDF), as a high-performance triboelectric material, exhibits great potential in TENGs owing to its unique polymorphic characteristics, superior electronegativity, and its β phase reflecting its piezoelectricity [2,3]. In recent years, numerous attempts have been made to enhance the triboelectric output performance of PVDF-based TENGs. For instance, the incorporation of inorganic nanofillers into PVDF during the electrospinning process has been demonstrated as an effective strategy to improve surface charge density and charge transfer efficiency [4,5,6]. However, due to the agglomeration tendency of nanoparticles, achieving uniform dispersion of inorganic nanofillers within PVDF spinning solutions remains challenging [7]. Additionally, the introduction of nanofillers necessitates corresponding optimization of process parameters during the electrospinning fabrication of PVDF composite nanofibers.

As a key additive for modifying the charge characteristics and charge generation rate of carbon powder, Charge Control Agents (CCAs) have been widely utilized in printer toner formulations since their early development. However, to the best of our knowledge, no relevant studies have been reported on the application of CCAs in TENGs until now. In this study, n-propyl gallate (PG), a negative-polarity CCA with excellent thermal stability and high solubility [8,9], was introduced during the electrospinning process of PVDF. The effects of trace amounts of PG on the characteristics of PVDF films were investigated. Various TENGs were fabricated using PVDF/PG composite nanofiber films. The triboelectric output characteristics of these devices were subsequently characterized under periodic mechanical movements, resulting in large enhancements in TENG output performance without affecting its β phase, and showcasing self-powered applications. This research not only provided a novel approach for preparing high-performance PVDF-based triboelectric layers but also offered deeper insights into the triboelectrification mechanism of PVDF.

## 2. Materials and Methods

### 2.1. Preparation of PVDF/PG Composite Nanofiber Films and Structural Design of PG-TENGs

To prepare the PVDF/PG nanofiber films, a measured amount of PVDF powder (with a particular molecular weight of 500,000, Yuanye Bio-Technology Co., Ltd., Shanghai, China) and PG (AR, Nanjing Dulai Bio-Technology Co., Ltd., Nanjing, China) were placed in a beaker. Subsequently, a specific volume of DMF (AR, Shanghai Aladdin Biochemical Technology Co., Ltd., Shanghai, China) and acetone (AR, Shanghai Lingfeng Chemical Reagent Co., Ltd., Shanghai, China) in a 3:2 ratio was added to the beaker containing the powder. The mixture was then stirred using a magnetic stirrer (AM-5250B, Tianjin Autoscience Instrument Co., Ltd., Tianjin, China) at 65 °C and 1000 rpm for 30 min to ensure complete dissolution of the powder. Following the stirring process, the solution was allowed to cool and age for 24 h. Finally, electrospinning solutions with PG additive amounts of 0.5 wt%, 1 wt%, 1.5 wt%, 2 wt%, and 2.5 wt%, and a PVDF concentration of 12.5 wt%, were obtained.

**Figure 1:**
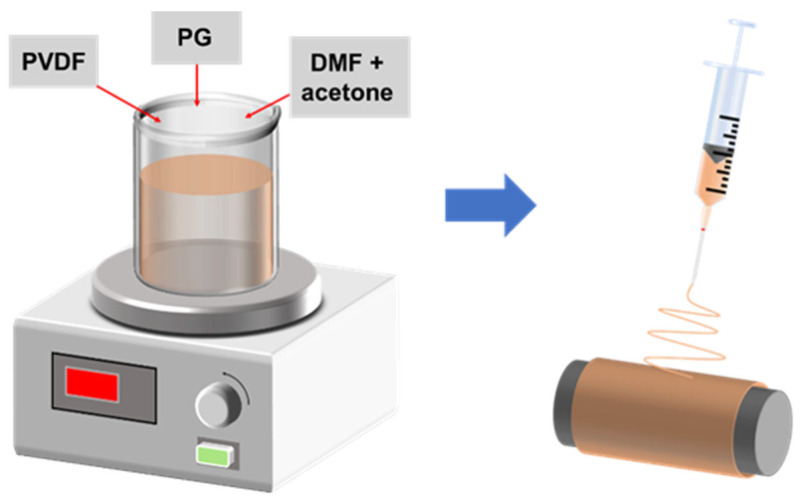
Schematic diagram of the preparation of PVDF/PG composite nanofiber films by electrospinning process shown on the right by using a syringe, after being stirred in a solution consisting of DMF+acetone together with PVDF and PG, as shown on the left.

The composite nanofiber films were fabricated using the electrospinning method. First, the prepared spinning solution was loaded into a 20 mL syringe with a needle inner diameter of 0.6 mm, which was then fixed to the corresponding position on a stepper motor. The electrospinning parameters were set as follows: spinning temperature of 45 ± 5 °C, relative humidity of 40 ± 5%, syringe feed rate of 1 mL/h, needle voltage of 18 kV, drum voltage of −2 kV, drum rotation speed of 450 rpm, and working distance of 15 cm. Aluminum tape was attached to the drum to collect the PVDF/PG nanofiber films. Following the electrospinning, the obtained PVDF/PG composite nanofiber films were subjected to heat treatment in a vacuum drying oven at 60 °C for 2 h. The schematic diagram is shown in Figure 1.

**Figure 2:**
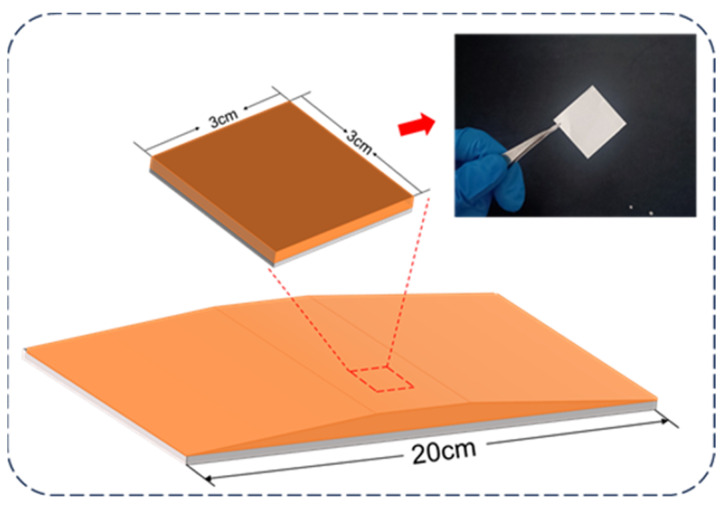
Photograph of PVDF/PG composite nanofiber films prepared for TENG device fabrication.

The properties of PVDF/PG composite nanofibers were characterized by using Fourier transform infrared spectroscopy (VERTEX 70, Bruker, Karlsruhe, Germany), X-ray diffraction (MiniFlex600, Rigaku, Tokyo, Japan), and field-emission scanning electron microscopy (ULTRA 55, Zeiss, Jena, Germany). Homogeneous thickness regions of the resulting films were subsequently sectioned into 3 cm × 3 cm square specimens for TENGs device fabrication, as shown in Figure 2. The TENG structure and fabrication methodology are comprehensively described in Supporting Information S1.

### 2.2. Measurement of the Electrical Properties of PG-TENGs

A series of electrical performance tests were conducted on the assembled PG-TENG. Since TENGs convert mechanical energy into electrical energy from the environment, it is necessary to apply constant force on the device for regular form of signal collection. For this purpose, a DC motor (TS-37GB555H-18, DongGuan Tsiny Motor Industrial Co., Ltd., Dongguan, China) with controllable frequency and force was used to apply vertical force onto the PG-TENG. The output performance was analyzed by using equipment such as an oscilloscope (DS2302A, RIGOL, Suzhou, China) and an electrometer (6517A, Keithley, Cleveland, OH, USA), as illustrated in Figures S1 and S2. Unless otherwise specified, the contact and separation motion frequency used in subsequent experiments was 2.7 Hz, and the applied pressure was 9 N.

## 3. Results and Discussion

### 3.1. Analysis of β-Phase Content and Surface Morphology of PVDF/PG Composite Nanofiber Films

The β-phase of PVDF/PG composite nanofiber films were studied by the Fourier transform infrared (FTIR) spectroscopy. According to relevant studies, the characteristic absorption peaks of the non-polar α-phase of PVDF films were observed at 764, 855, 975, 1148, and 1413 cm^−1^ in the FTIR spectra, while the characteristic absorption peaks of the polar β-phase were observed at 840, 1276, 1402, and 1431 cm^−1^ [10]. The β-phase content was calculated based on the following Beer–Lambert law [11]:

(1) $$F\left(\beta \right)=\frac{A_{\beta }}{K_{\beta }\;/K_{\alpha }A_{\alpha }+A_{\beta }}$$

Here, *A_α_* and *A_β_* represented the absorption intensities at 764 cm^−1^ and 840 cm^−1^, respectively, while *K_α_* and *K_β_* were the absorption coefficients, with values of 6.1 × 10^4^ and 7.7 × 10^4^ cm^2^/mol, respectively.

**Figure 3:**
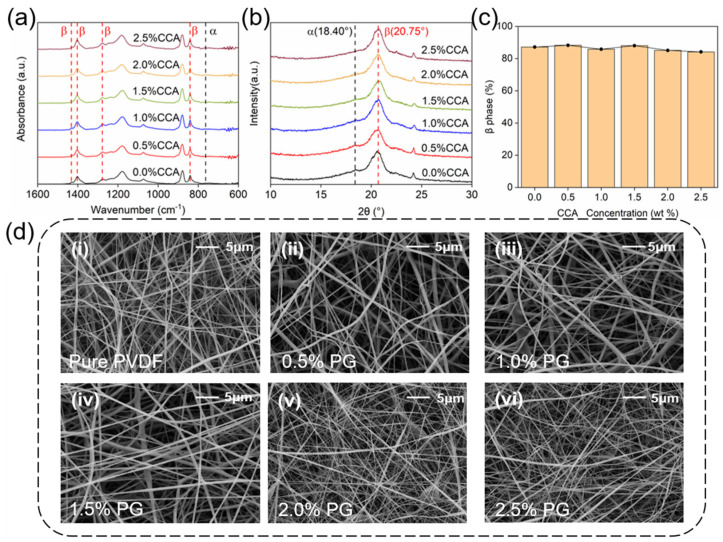
(**a**) FTIR analysis of PVDF/PG composite nanofiber films, (**b**) XRD analysis of PVDF/PG composite nanofiber films, (**c**) β-phase content in PVDF/PG composite nanofiber films (**d**) FESEM images of PVDF/PG nanofiber films.

The FTIR spectra of all the samples obtained via electrospinning are shown in Figure 3a. The results revealed that the characteristic absorption peaks of the non-polar α-phase are very weak, while the peaks corresponding to the polar β-phase are significantly more intense, as expected. The latter phase reflects the piezoelectricity of PVDF and can be enhanced thanks to the high electric field during electrospinning. During this process, the proportion of the β-phase of PVDF increases. Based on the Beer-Lambert law, the relative β-phase content of the pure PVDF nanofiber film was calculated to be 87.2%.

The diffraction peak corresponding to the (110) crystal plane of the β-phase in PVDF nanofiber films was located at 2θ = 20.75° by XRD, while a slight peak for the α-phase appears at 2θ = 18.4° [12], as seen in Figure 3b. The XRD patterns of all samples were highly consistent with each other, with negligible variations in peak positions and intensities. Thus, XRD results supported the fact that the addition of small amounts of PG did not significantly alter the relative β-phase content of the PVDF nanofiber films, implying no substantial impact on their piezoelectric properties, as can be seen in Figure 3c [13]. For PVDF composite nanofiber films with varying PG contents, the β-phase relative content remained at approximately 87%, which was comparable to that of pristine PVDF. Furthermore, FESEM images in Figure 3d demonstrate that the nanofibers exhibit a dense and uniform morphology with ultra-smooth surface topography. The average diameter of each sample fiber was about 370 ± 150 nm, measured by Nano Measure 1.2 software. The above results are in accordance with the fact that the incorporation of trace amounts of PG during the electrospinning process does not significantly affect the physicochemical properties of PVDF nanofiber films.

### 3.2. Electrical Performance of PG-TENGs

**Figure 4:**
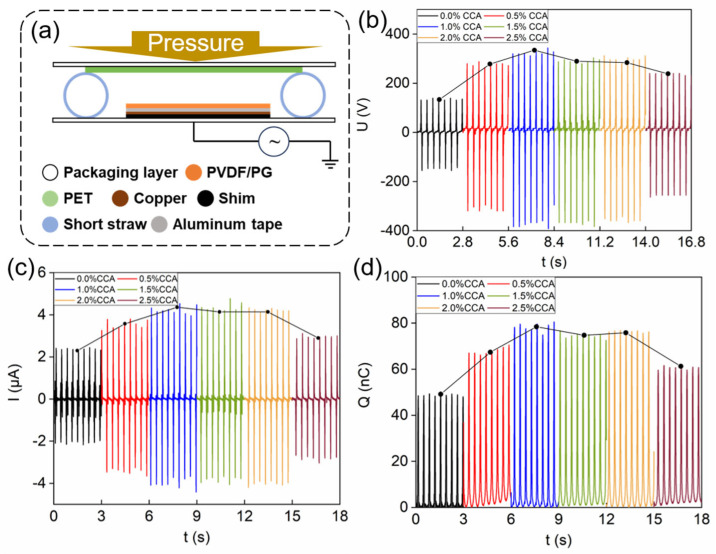
(**a**) Schematic illustration of the single-electrode mode PG-TENG structure, (**b**) output voltage of the single-electrode PG-TENG, (**c**) output current of the single-electrode PG-TENG, (**d**) transferred charge of single-electrode PG-TENG.

Using electrospun PG/PVDF nanofiber films, a series of PG-TENGs were fabricated and electrically characterized in the simplest TENG operation of single-electrode mode depicted in Figure 4a for quick and reliable comparison (see Supporting Information S2 for the working principle of this TENG). The experimental output performance of PG-TENGs was strongly correlated with the PG content, unlike their unvarying β-phase content. As shown in Figure 4b–d, the output voltage, current, and transferred charge of PG-TENGs with varying PG contents were systematically characterized. Notably, the PG-TENGs exhibited peak performance at a PG content of 1% (labeled as PG1-TENG, hereafter), achieving an output voltage of 334 V, an output current of 4.36 μA, and a transferred charge of 78.4 nC. Further increasing the PG content resulted in a decline in output performance; however, all PG-TENGs outperformed the pristine PVDF-TENG.

**Figure 5:**
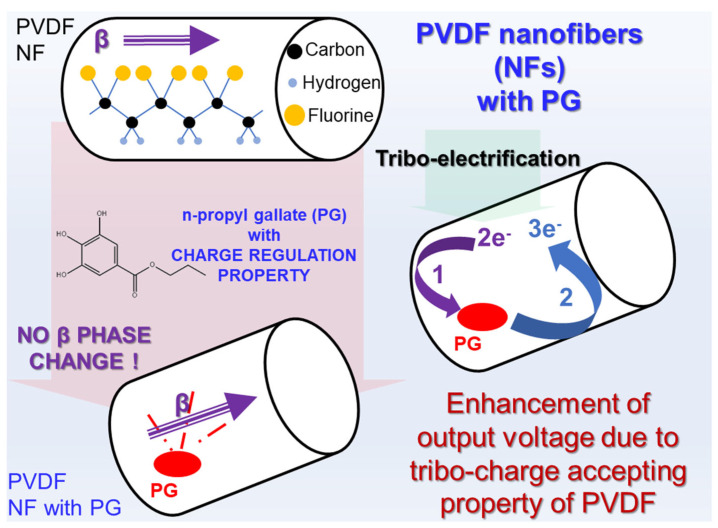
The tribo-charge enhancement mechanism of PVDF nanofibers with PG nanofiller.

This optimization could be attributed to the charge regulation ability of PG. PG is an ester with low effective acidity, which makes it act as inert within PVDF [14], and the β phase of PVDF stays unaffected, since PG is not involved in the structure of PVDF. Organic insulators have the property of acting as electron acceptors or donors, while PG, as a negative polarity CCA, has lower acceptor properties, which means that when it is charged by triboelectrification, PG acts as a donor and generates charges in free radicals [8]. Therefore, once tribo-charges enter the PG, they would bounce back into the PVDF matrix and carry additional charges, as illustrated in Figure 5. To be numerical, if 2 tribo-charges are accepted by PG, in return PG provides 3 tribo-charges, meaning it can generate an additional electron and supply that into the PVDF matrix.

The rationale behind the output enhancement of PG-1-based TENG without changing the β phase can be understood as follows: in experiments, the β-phase content in PVDF nanofiber films is solely attributed to the electrostatic stretching induced during the electrospinning process. The determination of the β-phase content is performed by a combination of FTIR and XRD measurements and analysis, which showed no considerable variation in the β-phase when we changed the PG concentration, which indicates that there is no effect of PG on the β-phase, confirming the hypothesis of no effect of inert PG in PVDF, as described above. In support of this, the systematic TENG performance studies clearly demonstrate that the addition of n-propyl gallate shows variation in the performance of the TENG, and for a specific concentration we were able to observe the best performance.

On a further note, since PG is an antioxidant, it could reduce the free radical content in PVDF and improve its antioxidant performance [15]. Studies have shown that tribo-charges can dissipate faster as the density of free radicals decreases [16]. So, when the PG content becomes too high, it would cause the dissipation of the stored tribo-charges within the PVDF nanofiber tribo-layer, resulting in a decrease in TENG output performance. This intriguing feature will be investigated in future studies of other CCA as fillers in PVDF nanofibers.

### 3.3. Exploration of Applications of PG-TENG

**Figure 6:**
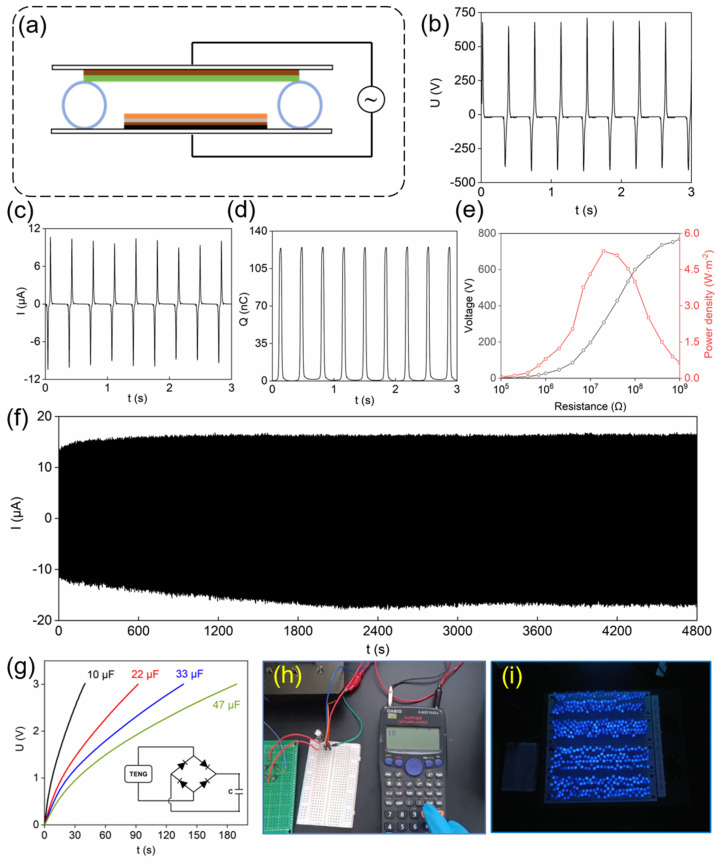
(**a**) Schematic illustration of the contact–separation mode PG-TENG, (**b**–**d**) Output voltage, current and transferred charge of dual-electrode PG1-TENG, (**e**) Power density of PG1-TENG under varied external resistances, (**f**) Output stability of PG1-TENG for ~13,000 cycles, (**g**) Charging curves of PG1-TENG toward different capacitors, (**h**) PG1-TENG powered LCD calculator, (**i**) 480 LEDs turned on by PG1-TENG.

Following the above experimental results, the PG1-TENG was operated in contact–separation mode, as described in Figure 6a, for the possibility of amplifying the signal for an energy-efficient self-powered system. The output voltage, current, and transferred charge were measured, as in Figure 6b–d; and indeed, a maximum output voltage of 712 V, with output current and transferred charge values of 10.66 μA and 125.3 nC, respectively, were obtained.

As this design allows the measuring of power density, resistors with different resistance values were connected to the PG1-TENG, for direct measurement of output power density. The curves of output voltage and power density as a function of external resistance were shown in Figure 6e. As observed, the output voltage increased with the external resistance, reaching its maximum value when the external resistance was 1 GΩ. Meanwhile, the output power density initially increased and then decreased with increasing external resistance, peaking at approximately 5.27 W/m^2^ when the external resistance was 20 MΩ. Table 1 shows the performance comparison between TENGs based on PVDF with different fillers and PG1-TENG. Figure 6f confirms the long-term stability of the device within ~13,000 cycles, with the output current amplitude of the device remaining constant. This stable operation is due to several facts: firstly, uniform dispersion of n-propyl gallate in the PVDF (SEM images show no agglomeration phenomenon), avoiding structural failure caused by phase separation; secondly, the antioxidant feature of the gallic acid structure of PG can reduce the charge attenuation caused by environmental factors; and finally, the structure of PG1-TENG is simple, not prone to deformation or damage during operation.

These long periods of operation hold promise for industrial applications, such as power equipment monitoring, which typically requires flexible sensors. The next challenge to tackle would be operation in harsh environments and obtaining long-term field performance data, which can make PG1 TENG, operating in contact–separation mode, suitably integrated in large electrical equipment.

Further, the energy harvesting capability of the PG1-TENG was evaluated by charging capacitors with different capacitance values. The charging curves in Figure 6g show the time required for the PG1-TENG to charge various capacitors to 3 V through a bridge rectifier circuit. As observed, larger capacitors required longer charging time. These results indicated that the PG1-TENG can be integrated with capacitors to drive low-power electronic devices. After charging a 470 μF capacitor for a while, the LCD calculator could be powered by the stored energy in the capacitor, as shown in Figure 6h and Video S1. Subsequently, Figure 6i and Video S2 reveal that 480 blue LEDs could be turned on by PG1-TENG under periodic mechanical excitations. These experimental results have provided some directions on how PG1-TENG can be utilized for real-time applications, as another example of PVDF commercialization. The contact–separation mode of TENG demonstrated the potential of PG1-TENG as a novel energy supply for self-powered wearable devices, Internet of Things sensors, emergency lighting systems, and so on. This can be realized if some current limitations can be overcome, such as TENG packaging and efficiency issues. The latter one is related to the energy storage of the external devices through capacitors. Both issues should be addressed in future studies by improving TENG structure and design, as well as materials’ properties as mentioned earlier.

## 4. Conclusions

In summary, a series of PVDF nanofiber films with varying PG contents were successfully prepared via electrospinning. Experimental techniques like FTIR, XRD and FESEM demonstrated that the incorporation of PG did not alter the β-phase of PVDF. Surprisingly, the performance of TENGs utilizing PVDF/PG nanofiber films as the tribo-layer was found to be strongly dependent on the PG content. With the addition of 1 wt% PG, the PG1-TENG achieved maximum output values of 334 V, 4.36 μA, and 78.4 nC for output voltage, current, and transferred charge, respectively, in single-electrode mode, highlighting ~60% improvement compared to pristine PVDF-TENG, which was attributed to charge regulation ability of PG. Additionally, a contact–separation mode PG1-TENG was developed to enhance the output performance further for high-performance self-powered applications.

## Figures and Tables

**Table 1:** Comparison of TENGs based on PVDF with various fillers.

TENGs	Triboelectric Pair	Output Voltage	Output Current	Max Power Density
P-TENG [17]	PVDF/PVC-TiO @ PET	235 V	35 μA	1.4 W/m^2^
EN-TENG [18]	PVDF/Mxene @ Nylon-11	270 V	24.7 μA	4.02 W/m^2^
NP-TENG [6]	PVDF/ZnSnO_3_ @ Al	138 V	5 μA	1.6 W/m^2^
ZIF-TENG [19]	PVDF/ZIF-67 @ FEP	395 V	95 μA	3.1 W/m^2^
BaTiO_3_-TENG [20]	PVDF/BaTiO_3_ @ Al	432 V	44.2 µA	2.25 W/m^2^
SA-TENG [21]	PVDF/SA-PTFE @ Silk	145 V	18 μA	2.6 W/m^2^
PG1-TENG (This work)	PVDF/PG @ PET	712 V	10.66 μA	5.27 W/m^2^

## Data Availability

The original contributions presented in this study are included in the article. Further inquiries can be directed to the corresponding authors.

## Supplementary Materials

The following supporting information can be downloaded at: https://www.mdpi.com/article/10.3390/ma18133089/s1, Figure S1: Schematic diagram of PG-TENGs testing setup; Figure S2: PG-TENGs electrical performance test platform; Figure S3: Photograph of PG-TENG; Figure S4: Working principle of PG-TENG; Supporting Information S1: Structural design and assembly of PG-TENG; Supporting Information S2: Working principle of PG-TENG; Video S1: Commercial LCD calculator power supply experiment; Video S2: LEDs lighting experiment.
