# Supplementary material for: High-Performance Triboelectric Nanogenerator Based on PVDF Nanofibers Modified by a Charge Control Agent n-Propyl Gallate
Source: Materials (Basel). 2025 Jun 30;18(13):3089. doi: 10.3390/ma18133089 (PMC12250774; doi:10.3390/ma18133089)
Supplement: Supplementary file 1 [file materials-18-03089-s001.zip › Supplementary Material.pdf]

Supporting Information

High-performance triboelectric nanogenerator based on PVDF nanofibers modified by a charge control agent n-propyl gallate

Chao Li [1], Xueying Yang [1], Xin Tang [1], Ying Yang [1], Linjiang Shen [1], Dawei Gu [1,*] and Mustafa Eginligil [2,*]

[1] Department of Physics, School of Physical and Mathematical Sciences, Nanjing Tech University, Nanjing 211816, China
2 Key Laboratory of Flexible Electronics (KLOFE) & Institute of Advanced Materials (IAM), Nanjing Tech University, Nanjing 3320239, China
* Correspondence: G: dwgu@njtech.edu.cn; Tel.: +86-13770736784
E: iameginligil@njtech.edu.cn; Tel.: +86-13505145217

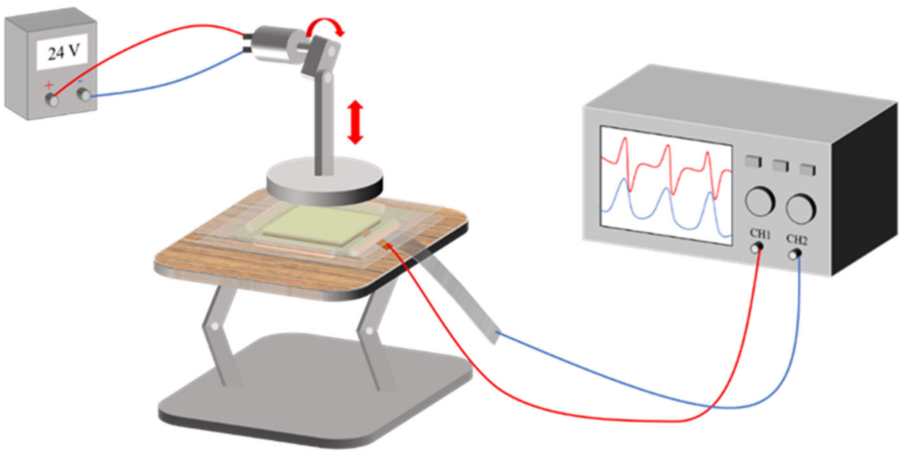

Figure S1. Schematic diagram of PG-TENGs testing setup.

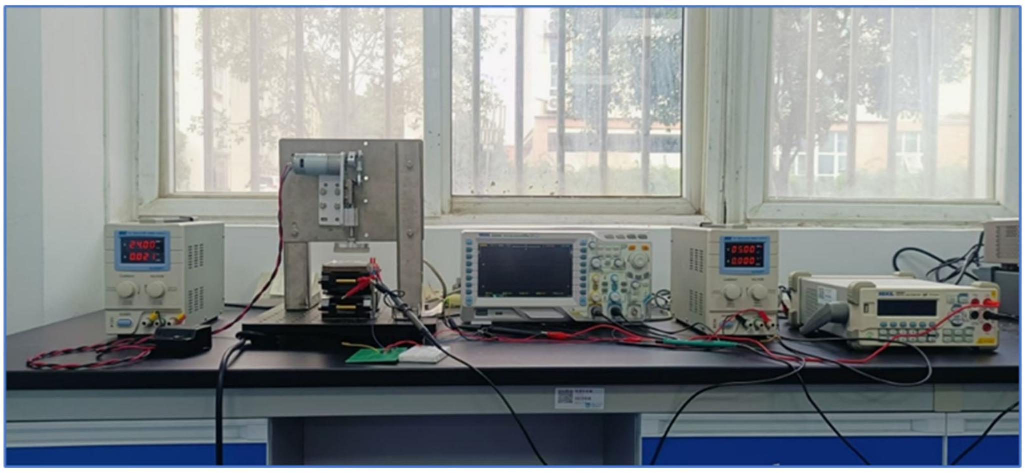

Figure S2. PG-TENGs electrical performance test platform.

Supporting Information S1. Structural design and assembly of PG-TENG

Experiment proposed a simple and cost-effective structure for the PG-TENG, utilizing a single-electrode operation mode. The negative triboelectric layer consisted of PVDF/PG composite nanofiber films, while the positive triboelectric layer was made out of PET plastic, which loses electrons when in contact with PVDF. The size of the PVDF/PG composite triboelectric layer was 3 cm × 3 cm. For the electrode, a copper foil of the same size was placed beneath the aluminum tape to connect to the external circuit. Four 3 cm-long straws were positioned around the PVDF triboelectric layer at a distance of approximately 0.5 cm, providing structural support and enabling automatic rebound after deformation of the TENG. To ensure sufficient contact between the upper- and lower-layers during friction, a 3 cm × 3 cm spacer was placed beneath the copper electrode, with a thickness equivalent to that of the flattened straws. The counter electrode was a 4 cm × 4 cm, 150 μm-thick transparent PET plastic sheet, placed directly above the PVDF triboelectric layer. Finally, the PVDF/CCA-TENG was encapsulated using two 10 cm × 7.5 cm transparent PVC cold laminating films to ensure structural stability.

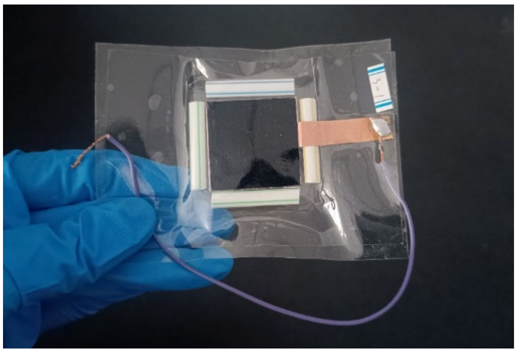

Figure S3. Photograph of PG-TENG

Supporting Information S2. Working principle of PG-TENG

The PVDF/PG composite nanofiber membrane served as the triboelectric layer, while PET was used as the counter electrode. Initially, when PVDF and PET were in close contact, PVDF, due to its stronger ability to capture negative charges, acquired negative triboelectric charges on its surface, while PET acquired an equal amount of positive triboelectric charges. At this stage, the PG-TENG was in an electrostatic equilibrium state (Fig. S5a). When PVDF and PET began to separate, the electrostatic equilibrium was disrupted, causing electrons on the copper electrode to flow to the ground through the wire due to electrostatic induction, resulting in a positive charge on the electrode. At this point, the PG-TENG exhibited an external current flow (Fig. S4b). When the distance between PVDF and PET reached a certain point, the system returned to electrostatic equilibrium, and the current dropped to zero (Fig. S4c). Subsequently, as PVDF and PET began to approach each other again, electrons flowed back to the electrode through the wire due to electrostatic induction until PVDF and PET were in close contact once more, restoring the system to electrostatic equilibrium (Fig. S4d). By repeating the contact-separation motion between PVDF and PET, the PG-TENG generated an alternating current output.

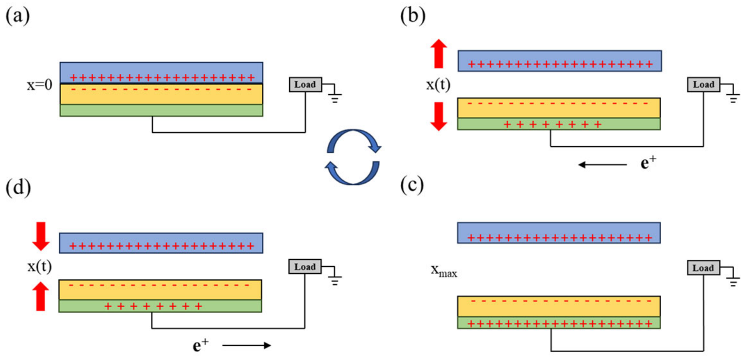

Figure S4. Working principle of PG-TENG
